# Hypoxia Induces Autophagy through Translational Up-Regulation of Lysosomal Proteins in Human Colon Cancer Cells

**DOI:** 10.1371/journal.pone.0153627

**Published:** 2016-04-14

**Authors:** Ming-Chih Lai, Chiao-May Chang, H. Sunny Sun

**Affiliations:** 1 Department of Biomedical Sciences, Chang Gung University, Taoyuan, Taiwan; 2 Department of Obstetrics and Gynecology, Chang Gung Memorial Hospital, Taoyuan, Taiwan; 3 Bioinformatics Center, National Cheng Kung University, Tainan, Taiwan; 4 Institute of Molecular Medicine, National Cheng Kung University, Tainan, Taiwan; School of Medicine, University of Belgrade, SERBIA

## Abstract

Hypoxia occurs in a wide variety of physiological and pathological conditions, including tumorigenesis. Tumor cells have to adapt to hypoxia by altering their gene expression and protein synthesis. Here, we showed that hypoxia inhibits translation through activation of PERK and inactivation of mTOR in human colon cancer HCT116 cells. Prolonged hypoxia (1% O_2_, 16 h) dramatically inhibits general translation in HCT116 cells, yet selected mRNAs remain efficiently translated under such a condition. Using microarray analysis of polysome- associated mRNAs, we identified a large number of hypoxia-regulated genes at the translational level. Efficiently translated mRNAs during hypoxia were validated by polysome profiling and quantitative real-time RT-PCR. Pathway enrichment analysis showed that many of the up-regulated genes are involved in lysosome, glycan and lipid metabolism, antigen presentation, cell adhesion, and remodeling of the extracellular matrix and cytoskeleton. The majority of down-regulated genes are involved in apoptosis, ubiquitin-mediated proteolysis, and oxidative phosphorylation. Further investigation showed that hypoxia induces lysosomal autophagy and mitochondrial dysfunction through translational regulation in HCT116 cells. The abundance of several translation factors and the mTOR kinase activity are involved in hypoxia-induced mitochondrial autophagy in HCT116 cells. Our studies highlight the importance of translational regulation for tumor cell adaptation to hypoxia.

## Introduction

Colorectal cancer (CRC) is one of the most common cancers in humans. Every year, more than 1 million patients are diagnosed with CRC in the world. The incidence of CRC has been rising steadily in the last 20 years [1]. Studies of CRC have provided valuable insights into the multistep genetic process of carcinogenesis [2, 3]. The majority of CRC is triggered by mutations in adenomatous polyposis coli (*APC*) gene [4], which induces the formation of early adenoma. The development of colon cancer is further promoted by a series of genetic mutations, including *KRAS*, *SMAD4*, and *TP53*, which enable adenoma growth and carcinoma progression. A better understanding of the molecular mechanisms underlying CRC progression may facilitate the development of new anti-cancer therapeutics. Over the past decade, several new molecular targets are currently being investigated for the treatment of CRC [5].

Because of rapid proliferation and aberrant angiogenesis, tumors contain areas with various degrees of hypoxia [6]. Tumor cells have to adapt to hypoxic stress by altering their gene expression and protein synthesis [7]. These alterations include energy metabolism, angiogenesis, cell migration, tumor invasion and metastasis, cell cycle regulation, inflammatory response, and pH regulation [8–10]. Tumor hypoxia is thought to play a key role in tumor progression and malignancy. The presence of hypoxic cells in solid tumors is associated with a poor prognosis in many types of cancers [6]. Previous studies have shown that hypoxic tumor cells are more resistant to radiotherapy [11, 12] and many commonly used chemotherapeutic agents [13]. Therefore, it is worthwhile to investigate the regulation of gene expression in human cancer cells under hypoxic conditions.

In response to hypoxia, cells rapidly increase the level of hypoxia-inducible factor 1 (HIF-1) [14, 15], a heterodimer composed of an oxygen-sensitive HIF-1α subunit and a constitutively expressed HIF-1β subunit. HIF-1 regulates oxygen homeostasis during hypoxia by transcriptionally targeting more than 100 genes, which contain hypoxia-responsive elements (HREs) within their promoters [16, 17]. In addition to transcription, translation is regarded as an important contributor to hypoxia-regulated gene expression [18, 19]. Metabolic labeling studies showed that hypoxia inhibits global translation in many different cell lines [20–23]. General translation is significantly inhibited by acute anoxia (<0.02% O_2_) or prolonged hypoxia (≦2% O_2_, >16 h) [21, 22, 24–26]. It has been reported that mammalian target of rapamycin (mTOR) and the endoplasmic reticulum resident kinase (PERK) play key roles in translational regulation during hypoxia [26–28]. Hypoxia inhibits the kinase activity of mTOR and leads to dephosphorylation of translation initiation factor 4E-binding proteins (4E-BPs). Dephosphorylation of 4E-BPs increases their binding affinity to the translation initiation factor eIF4E and thus suppresses cap-dependent translation by disrupting association of eIF4E with eIF4G. On the other hand, hypoxia induces the unfolded protein response (UPR), which occurs as a consequence of endoplasmic reticulum (ER) stress, and leads to the activation of PERK [21, 24, 27]. Activated PERK phosphorylates eukaryotic translation initiation factor 2 subunit α (eIF2α) and thus inhibits translation initiation by preventing the formation of the eIF2-GTP-tRNA(i)Met ternary complex [29].

Although general translation is largely inhibited during hypoxia, selected mRNAs remain efficiently translated under such a condition. Translation of hypoxia-responsive genes requires alternative mechanisms, such as internal ribosome entry site (IRES) [30, 31] and upstream open reading frame (uORF) [32]. IRES is a complex RNA structural element that initiates translation by directly recruiting ribosomes to find the start codon on an mRNA. It is thought that hypoxia represses cap-dependent but not IRES-mediated translation initiation [29]. Hypoxia-responsive genes HIF-1α and VEGFA have been shown to maintain translation through IRES during hypoxia [33, 34]. However, the mechanism of translational regulation during hypoxia is still not fully understood, and it may vary depending on the cell types and hypoxic conditions.

Autophagy is a cell biological process that degrades unnecessary or dysfunctional proteins and organelles to maintain nutrient and energy homeostasis during stress conditions [35]. It has been reported that hypoxia induces autophagy in a HIF-1-dependent manner [36, 37]. However, translational regulation also plays an important role in hypoxia-induced autophagy. In this study, we used polysome profiling coupled to whole human genome expression array to identify candidate genes whose translation is regulated by hypoxia in human colon cancer HCT116 cells. Functional annotation of candidate genes indicates that hypoxia regulates translation of a lot of genes involved in lysosome and different metabolic pathways. We provide evidence that hypoxia induces autophagy through translational up-regulation of lysosomal proteins in HCT116 cells. Our studies emphasize the importance of translational regulation for tumor cell adaptation to hypoxia.

## Materials and Methods

### Cell culture and hypoxic treatment

HCT116 cells (ATCC^®^ CCL247^™^) were grown in MEM medium supplemented with 10% fetal bovine serum at 37°C in 5% CO_2_ incubator. For hypoxic (1% O_2_) treatment, cells were transferred to a specially designed hypoxia chamber (NexBiOxy Inc., Hsinchu, Taiwan) which was flushed with 95% N_2_ and 5% CO_2_ at 37°C.

### Plasmids and transfection

The plasmids expressing FLAG-tagged wild type mTOR or constitutively active mutants (L1460P & E2419K) were purchased from Addgene (Cambridge, MA). Cell transfection was performed using the transfection reagent Lipofectamine^®^ 2000 (Thermo Fisher Scientific), essentially according to the manufacturer’s instructions.

### RNAi-mediated knockdown

All of the plasmids required for RNAi-mediated knockdown were provided by the National RNAi Core Facility (Academia Sinica, Taiwan). The two pLKO.1-shRNA vectors used to knockdown PSAP and LAMP2 were as follows: TRCN0000217974 (shPSAP), and TRCN0000029263 (shLAMP2). The transfection reagent Lipofectamine^®^ 2000 (Thermo Fisher Scientific) was used to transfer plasmid DNA into HCT116 cells. Cells were harvested 3 days post-transfection for analysis.

### Sucrose gradient sedimentation and polysome profiling

Cells were collected in cold PBS containing 100 μg/ml cycloheximide. All subsequent steps were performed at 4°C. Cell pellets were resuspended in RSB-150 (10 mM Tris-HCl (pH 7.4), 3 mM MgCl_2_, and 150 mM NaCl) containing 100 μg/ml cycloheximide, 40 μg/ml digitonin (Calbiochem), 20 U/ml RNasin (Promega), and 1X protease inhibitor cocktail (Thermo Fisher Scientific). After incubation on ice for 5 min, cells were disrupted by passage through a 26-gauge needle five times. Cytoplasmic extracts were collected by centrifugation at 3,000 × g for 2 min, and clarified by further centrifugation at 11,000 × g for 15 min. The samples were loaded on a linear 15–40% sucrose gradient and centrifuged at 38,000 rpm for 3 h in a Beckman SW41 rotor. After centrifugation, total RNA was extracted from each fraction using phenol/chloroform extraction in the presence of 1% SDS and 0.25 M NaCl, followed by ethanol precipitation. For polysome profile analysis, the gradients were monitored at 254 nm using an ISCO fractionation system (Lincoln, NE).

### Immunoblotting

Proteins were transferred onto a PVDF Transfer Membrane (PerkinElmer). Protein blots were blocked with 3% skim milk in TBST buffer (100 mM Tris-HCl (pH 7.6), 150 mM NaCl, and 0.05% Tween 20) at RT for 1 h. The primary antibodies included rabbit anti-phospho-eIF2α (Ser51) (1:1000 dilution; Cell Signaling), mouse anti-eIF2α (0.4 μg/ml; Santa Cruz Biotechnology), rabbit anti-phospho-4E-BP1 (Thr37/46) (1:1000 dilution; Cell Signaling), mouse anti-4E-BP1 (0.4 μg/ml; Santa Cruz Biotechnology), mouse anti-β-actin (1:2500 dilution; Sigma-Aldrich), mouse anti-HIF-1α (0.2 μg/ml; BD Transduction Laboratories), goat anti-Glut1 (1 μg/ml; Santa Cruz Biotechnology), rabbit anti-VEGF (0.4 μg/ml; Santa Cruz Biotechnology), rabbit anti-LC3B (1:1000 dilution; Cell Signaling), rabbit anti-p62 (1:2000 dilution; MBL), rabbit anti-α-tubulin (1:2000 dilution; Cell Signaling), rabbit anti-GNS (1:200 dilution; GeneTex), rabbit anti-PSAP (1:1000 dilution; GeneTex), mouse anti-TPP1 (1 μg/ml; Abcam), mouse anti-ATPB (0.5 μg/ml; Abcam), rabbit anti-LAMP2 (1:1000 dilution; GeneTex), rabbit anti-eIF4E (1 μg/ml; Abcam), and rabbit anti-eIF4A1 (1 μg/ml; Abcam). Blots were incubated with primary antibodies in blocking buffer at RT for 2 h, followed by incubation with HRP-conjugated secondary antibodies at RT for 2 h. Detection was performed using Immobilon Western Chemiluminescent HRP Substrate (Millipore) for X-ray film exposure.

### Microarray analysis

For microarray analysis, isolated RNA was cleaned up with the RNeasy Mini kit (Qiagen). Approximately 6 μg of total RNA was reverse transcribed into cDNA using an oligo d(T) primer that contains the T7 RNA polymerase promoter sequence at the 3’ end. Biotin-labeled complementary RNA (cRNA) was produced by *in vitro* transcription followed by metal-induced hydrolysis at 94°C. Subsequently, fragmented cRNA was hybridized onto Affymetrix Human Genome U133 Plus 2.0 Array at 45°C for 16 h. Subsequent washing and staining were performed with a Fluidic Station-450 and GeneChips are scanned with Affymetrix GeneChip Scanner 7G. Raw microarray data were further analyzed using GeneSpring GX 10 software (Silicon Genetics).

### RT-PCR and quantitative real-time PCR

RT-PCR was used to detect the mRNA expression level. Extracted RNA was reverse-transcribed into cDNA using the High-Capacity cDNA Reverse Transcription Kits (Thermo Fisher Scientific) according to manufacturer’s instructions. The resulting cDNA was subjected to conventional PCR or quantitative real-time PCR analysis. Conventional PCR was performed using GoTaq DNA polymerase (Promega) and the forward and reverse primers: β-actin (forward primer (FP): 5’CATCCACGAAACTACCTTCAACT3’ and reverse primer (RP): 5’TCTCCTTAGAGAGAAGTGGGGTG3’), HIF-1α (FP: 5’TGGACTCTGATC ATCTGACC3’ and RP: 5’CTCAAGTTGCTGGTCATCAG3’), and VEGFA (FP: 5’CCTGGT GGACATCTTCCAGGAGTACC3’ and RP: 5’GAAGCTCATCTCTCCTATGTGCT GGC3’) [38].

Quantitative real-time PCR was performed using StepOnePlus^™^ Real-Time PCR Systems according to suppliers' recommendations (Thermo Fisher Scientific). Primers used for quantitative real-time PCR are listed in S1 Table. The levels of mRNAs were detected with Fast SYBR^®^ Green Master Mix (Thermo Fisher Scientific). Quantitative analysis was performed by the measurement of CT values during the exponential phase of amplification. Relative quantitation values were calculated using the 2^-ΔΔCT^ method [39].

### Pathway enrichment analysis

Functional annotation of hypoxia-regulated genes was performed using the publicly available DAVID Bioinformatics Resources Version 6.7 software (http://david.abcc.ncifcrf.gov/) with the Kyoto Encyclopedia of Genes and Genomes (KEGG) pathway database. The statistical significance was assessed by a modified Fisher's exact test. P-value = 0 represents perfect enrichment. P-value <0.05 is considered strongly enriched in the annotation categories.

### Flow cytometry analysis

Cell cultures were vitally stained with acridine orange (AO; Sigma-Aldrich) at a concentration of 5 μg/ml for 15 min and then washed with PBS. AO is a fluorescent cationic dye that can be employed to measure the levels of acidic vesicular organelles (lysosomes) within cells. The levels of acidic vesicular organelles were analyzed using the FACSCalibur (BD Biosciences) with excitation set at 488 nm, and emission from fluorescent AO was detected by FL-3 channel (670 nm).

The mitochondrial membrane potential was determined using tetramethylrhodamine methyl ester (TMRM). Cell cultures were vitally stained with 0.5 μM TMRM for 30 min at 37°C and then washed with PBS. The fluorescence intensity of TMRM was analyzed using flow cytometry with FL-2 channel (564~606 nm). Samples were analyzed using CellQuest Pro 4.0.2 software (BD Biosciences) and quantification was performed using WinMDI 2.9 software (Scripps Research Institute, La Jolla, CA, USA).

### LysoTracker staining

The LysoTracker Red DND-99 (Thermo Fisher Scientific) is used to investigate the biosynthesis and accumulation of lysosomes according to the manufacturer’s instructions. Briefly, the HCT116 cells grown on cover slips were labeled with LysoTracker Red DND-99 (1:5000 dilution) for 1 h under growth conditions. After labeling, cells were washed with cold PBS and fixed with 3% formaldehyde in PBS for 30 min. After extensive washing with PBS, the specimens were mounted immediately and observed using an inverted fluorescence microscope (Zeiss Axio Observer A1, Germany) equipped with a CCD camera.

### Statistical analysis

All data were presented as mean ± standard error and analyzed using GraphPad Prism 4.0 software (GraphPad Software Inc., La Jolla, CA, USA). Statistical analysis was performed using an unpaired t-test. P-value <0.05 was considered statistically significant.

## Results

### General translation is susceptible to hypoxia in HCT116 cells

**Fig 1:**
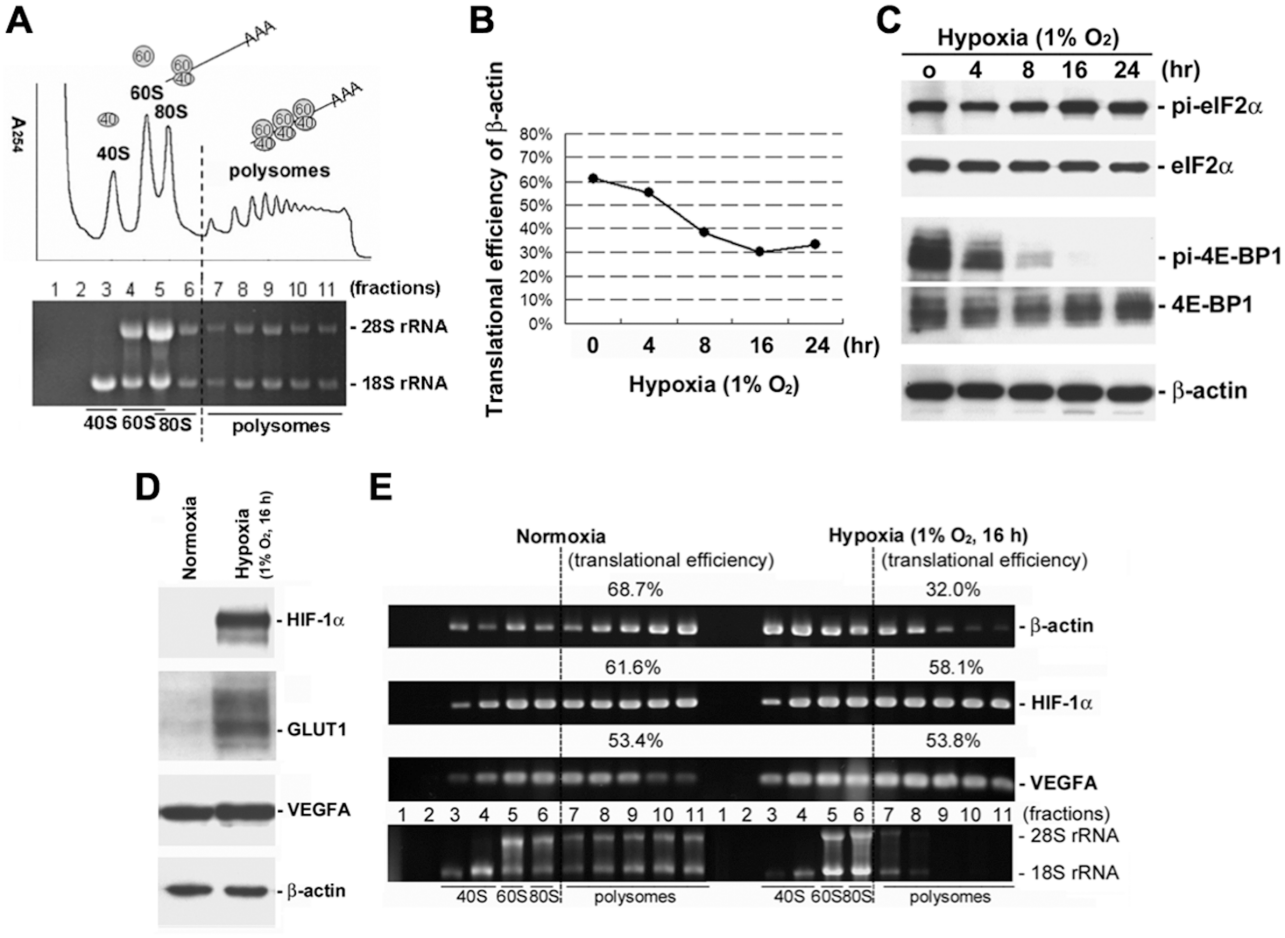
General translation is inhibited by hypoxia in HCT116 cells. **A**. Cytoplasmic extracts were loaded on a linear 15–40% sucrose gradient ultracentrifugation. After centrifugation, the polysome profile was plotted by A_254_ values (upper), and RNA was extracted from each fraction for analysis. The purified RNA was resolved on a 1% formaldehyde/agarose gel, and rRNA was visualized by ethidium bromide staining (lower). The distribution of ribosomal subunits and polysomes are indicated. **B**. HCT116 cells were treated with hypoxia (1% O_2_) for 0, 4, 8, 16, and 24 h. The polysomal distribution of β-actin mRNA was detected by polysome profiling and RT-PCR. Translational efficiency of β-actin mRNA was calculated and shown as a percentage at different time points. **C**. The phosphorylation status of eIF2α and 4E-BP1 was determined by immunoblot analysis in HCT116 cells exposed to hypoxia for the indicated period of time. The levels of phosphorylated (pi-) and total proteins were detected by specific antibodies against phospho-eIF2α (Ser51), eIF2α, phospho-4E-BP1 (Thr37/46), and 4E-BP1. Detection of β-actin protein served as a loading control. **D**. Immunoblot analysis of HIF-1α, GLUT1, VEGFA, and β-actin proteins in HCT116 cells exposed to hypoxia for 16 h. Detection of β-actin protein served as a loading control. **E**. HCT116 cells were grown under normoxia (21% O_2_) or hypoxia (1% O_2_) for 16 h. Cells were harvested and lysed in RSB-150 buffer. Cytoplasmic extracts were loaded on a linear 15–40% sucrose gradient ultracentrifugation and collected into 11 fractions (1 ml/fraction). RNA isolated from each fraction was detected by RT-PCR and subjected to agarose gel electrophoresis. The polysomal region of the gradient includes fractions 7–11. Translational efficiency of β-actin, HIF-1α, and VEGFA mRNAs was calculated and shown as a percentage. 28S and 18S rRNAs were directly visualized by ethidium bromide staining. The distribution of ribosomal subunits and polysomes are indicated.

It has been reported that general translation is inhibited by hypoxia in various cell types, but hypoxia-responsive genes can escape from translational repression during hypoxia [32–34, 40]. Here, we performed polysome profiling and RT-PCR to detect polysomal distribution of specific mRNAs. The mRNA/ribosome complexes were separated into 11 fractions using a linear 15–40% sucrose gradient centrifugation (Fig 1A). The distribution of an mRNA within the polysomal fractions is reflective of its translational efficiency. First, we used different colorectal cancer cell lines to study the effects of hypoxia on translation and observed that prolonged hypoxia (1% O_2_, ≧16 h) caused a dramatic inhibition of general translation in HCT116 cells (S1 Fig). Translational efficiency of housekeeping gene β-actin changes from ~60% to ~30% within 16 h of exposure to hypoxia (Fig 1B). Because PERK and mTOR kinases have been proposed as important factors in translational regulation during hypoxia [27], we therefore analyzed the phosphorylation status of their downstream substrates, eIF2α and 4E-BP1, in HCT116 cells under hypoxia. Immunoblot analysis showed that prolonged hypoxia leads to a slight increase in eIF2α phosphorylation (Fig 1C), and 4E-BP1 is gradually dephosphorylated within 16 h of exposure to hypoxia in HCT116 cells. This indicates that hypoxia inhibits general translation at least in part through activation of PERK and inactivation of mTOR in HCT116 cells.

To investigate changes in translation during hypoxia, HCT116 cells were grown under normoxic (21% O_2_) and hypoxic (1% O_2_) culture conditions for 16 h. As expected, hypoxia stabilizes HIF-1α protein and induces the expression of HIF-1 target genes GLUT1 and VEGFA in HCT116 cells (Fig 1D). We also performed polysome profiling and RT-PCR to evaluate translational efficiency of selected mRNAs. Denaturing agarose gel electrophoresis of rRNA showed a shift of mRNAs from polysomes into translation initiation complexes (Fig 1E, lower panel). The accumulation of 18S and 28S rRNAs in low molecular weight ribosomal fractions (fractions 5–6; hypoxia) indicated translational repression during hypoxia. The polysomal distribution of β-actin mRNA was evidently decreased in hypoxic HCT116 cells as compared to normoxic control (Fig 1E, upper panel). A large portion of the β-actin mRNA (68.7%) was associated with polysomes in normoxic HCT116 cells, whereas only 32.0% of β-actin mRNA remained associated with polysomes in hypoxic HCT116 cells. In contrast, the polysomal distribution of HIF-1α and VEGFA mRNAs were relatively unaffected by hypoxia (Fig 1E, middle panels). More than half of HIF-1α (58.1%) and VEGFA (53.8%) mRNAs remained associated with polysomes after 16 h exposure to hypoxia. In agreement with previous studies [33, 34], HIF-1α and VEGFA mRNAs have the ability to escape translational repression during hypoxia. Therefore, we assume that selected mRNAs remain efficiently translated in HCT116 cells under hypoxic conditions.

### Translational regulation of selected mRNAs in HCT116 cells during hypoxia

**Fig 2:**
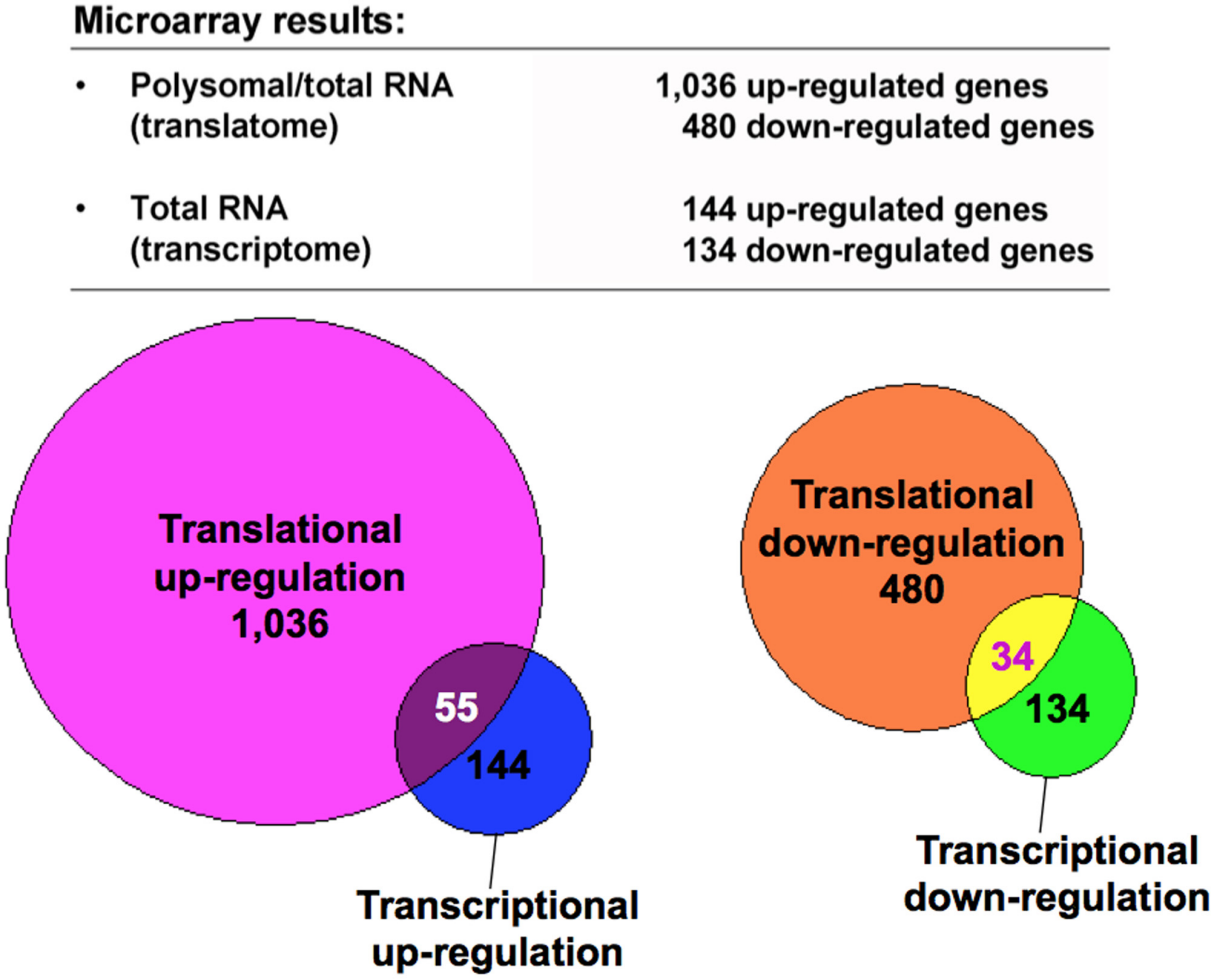
Identification of hypoxia-regulated genes in HCT116 cells. Results of microarray analysis were analyzed using GeneSpring GX 10 software. Genes with ≧2-fold change in the polysomal/total RNA ratio or total RNA were defined as hypoxia-regulated genes. The results were obtained from three independent experiments. Hypoxia-regulated genes were divided into four categories: up-regulated and down-regulated genes at the translational (translatome) and transcriptional (transcriptome) levels, respectively. Venn diagrams show the overlap of hypoxia-regulated genes between translatome and transcriptome. Numbers in overlapping areas indicate hypoxia-regulated genes at both the translational and transcriptional levels in HCT116 cells.

To investigate the impact of hypoxia on translation, we exploited polysome profiling coupled to cDNA microarray analysis to screen hypoxia-regulated genes. Both polysome-associated mRNAs and total RNA of normoxic and hypoxic HCT116 cells were isolated and subjected to microarray hybridization. Polysome-associated mRNAs were isolated from a pool of polysomal fractions (Fig 1A, fractions 8–11) for the analysis of translatome. Total RNA was extracted from the same samples for the analysis of transcriptome. The translational change of an mRNA was measured by the change in the abundance of polysomal RNA normalized to the change in the abundance of total RNA for each mRNA (Fig 2, polysomal/total). Genes with changes ≧2-fold after exposure to hypoxia were considered as hypoxia-regulated candidate genes. All candidate genes were divided into four categories: up-regulated and down-regulated genes at either the translational or transcriptional level (Fig 2). As a result, 1,036 up-regulated genes and 480 down-regulated genes were identified by translatome analysis. The parallel transcriptome analysis identified 144 up-regulated genes and 134 down-regulated genes. However, only ~32% of hypoxia-regulated genes at the transcriptional level exhibit signs of translational co-regulation in HCT116 cells during hypoxia (Fig 2, Venn diagrams). This indicates that hypoxia may affect different subsets of target genes between translatome and transcriptome.

### Validation of candidate genes whose translation is up-regulated by hypoxia in HCT116 cells

**Fig 3:**
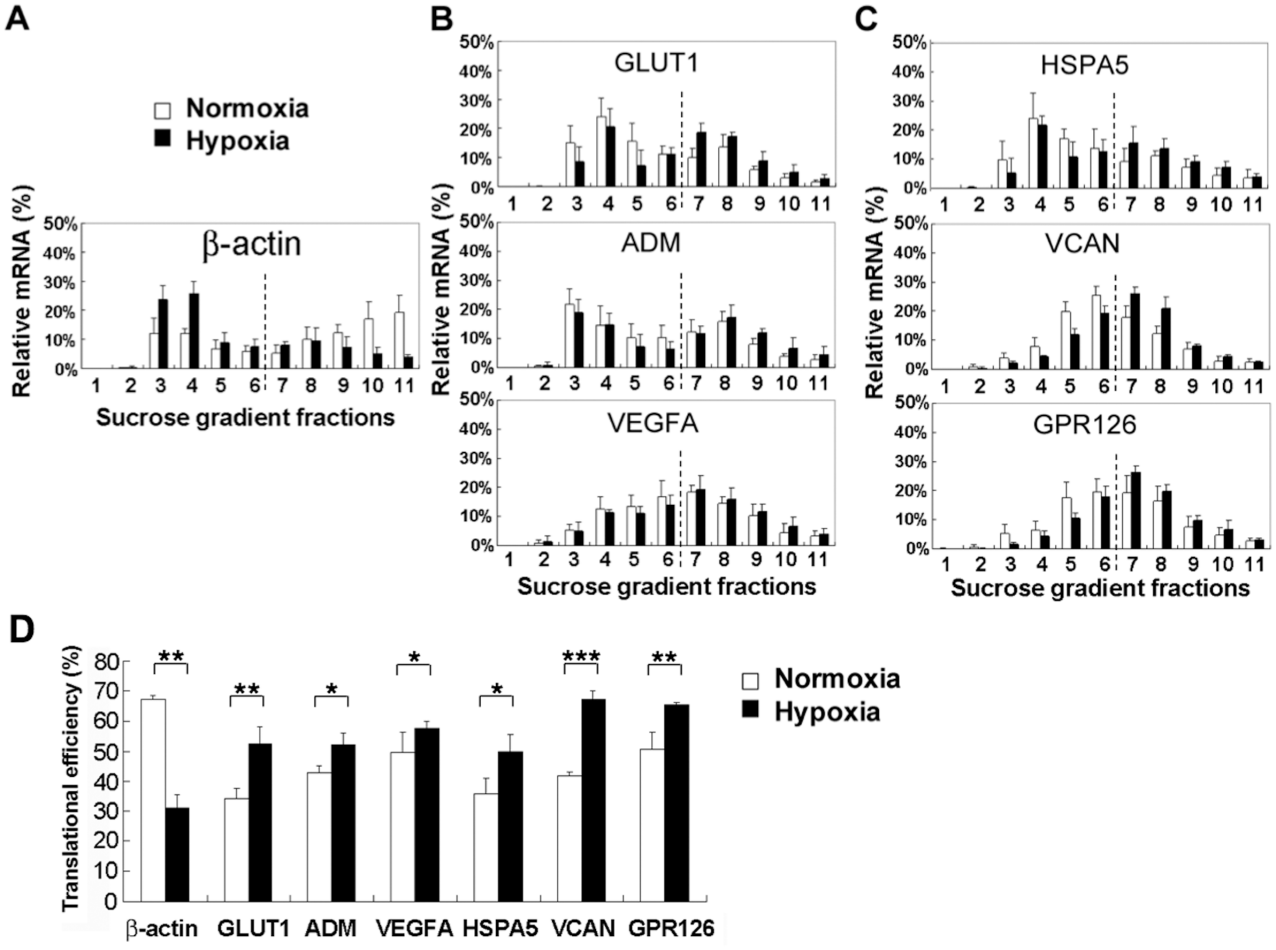
Validation of microarray results. Several up-regulated genes at the translational level (translatome) in hypoxic HCT116 cells were validated. RNA isolated from sucrose gradient fractionation was analyzed by quantitative real-time RT-PCR. The distribution of mRNAs in each fraction was calculated and shown as a percentage (%). **A**. Polysomal profile of β-actin served as a negative control. **B**. Polysomal profiles of up-regulated genes at both the translational and transcriptional levels (*GLUT1*, *ADM*, and *VEGFA*). **C**. Polysomal profiles of up-regulated genes at the translational but not transcriptional level (*HSPA5*, *VCAN*, and *GPR126*). **D**. Translational efficiency of β-actin, GLUT1, ADM, VEGFA, HSPA5, VCAN, and GPR126 mRNAs was calculated and shown as a percentage (%) in HCT116 cells under normoxia and hypoxia. Bar graphs show mean ± standard error from at least three independent experiments (*p < 0.05, **p < 0.01, ***p < 0.001).

To verify microarray data, several candidate genes were analyzed by polysome profiling and quantitative real-time RT-PCR. The polysomal association of β-actin mRNA was evidently decreased in HCT116 cells exposed to hypoxia (Fig 3A). In contrast, the polysome-associated mRNAs of both translationally and transcriptionally up-regulated genes *GLUT1*, *ADM*, and *VEGFA* were increased in HCT116 cells during hypoxia as compared to normoxia (Fig 3B), indicating that the three genes remain efficiently translated under hypoxia. Similar results were obtained from translationally but not transcriptionally up-regulated genes *HSPA5*, *VCAN*, and *GPR126* (Fig 3C). After calculation, these translationally up-regulated genes showed an increase in translational efficiency during hypoxia as compared to normoxia (Fig 3D). The results of validation experiments are largely consistent with microarray measurements. This indicates that many genes can escape from translational repression and remain efficiently translated in HCT116 cells during hypoxia.

### Pathway enrichment analysis of hypoxia-regulated genes at the translational level

In order to gain insight into the biological functions of hypoxia-regulated genes, pathway enrichment analysis was performed using the DAVID Bioinformatics Resources 6.7 software to map genes to biological pathways as defined by the KEGG pathway database. The results showed that hypoxia leads to translational up-regulation of genes that function in lysosome, glycan and lipid metabolism, antigen processing and presentation, cell adhesion, and remodeling of the extracellular matrix (ECM) and cytoskeleton (Table 1). In contrast, hypoxia down-regulates translation of genes involved in apoptosis, ubiquitin-mediated proteolysis, and oxidative phosphorylation (Table 2). A major function of lysosomes is the digestive autophagy in eukaryotic cells. Therefore, we assume that hypoxia induces lysosomal autophagy and metabolic rearrangement to maintain energy homeostasis via a translational mechanism.

**Table 1:** Functional classification of the translationally up-regulated genes in HCT116 cells exposed to hypoxia for 16 h.

KEGG_pathway	P-value	Genes
Lysosome	2.96E-13	*CTSL2*, *AP1G1*, *LGMN*, *ATP6AP1*, *HEXA*, *HEXB*, *PPT1*, *CTSA*, *CLTC*, *CTSL1*, *ATP6V0B*, *IDS*, *TPP1*, *SCARB2*, *MAN2B1*, *AP3B1*, *LAPTM4B*, *LIPA*, *LAPTM4A*, *PSAP*, *GUSB*, *CD164*, *CD63*, *M6PR*, *MANBA*, *GNS*, *LAMP1*, *LAMP2*, *NPC1*, *GAA*, *SORT1*, *CTSC*, *CTSB*, *CTSH*, *CLN5*
N-Glycan biosynthesis	5.31E-09	*B4GALT1*, *B4GALT3*, *RFT1*, *GANAB*, *MAN1B1*, *ALG5*, *ALG6*, *MAN1A1*, *ALG8*, *ALG9*, *MAN2A2*, *STT3B*, *MAN2A1*, *STT3A*, *RPN1*, *RPN2*, *DDOST*, *UTP14C*
Antigen processing and presentation	1.42E-05	*HSP90AA1*, *PDIA3*, *LGMN*, *HLA-A*, *HSPA1A*, *HLA-C*, *NFYA*, *HLA-B*, *HLA-E*, *CALR*, *CANX*, *CTSL1*, *HLA-G*, *B2M*, *HLA-F*, *TAPBP*, *TAP1*, *HSPA4*, *HSPA5*, *CTSB*
ECM-receptor interaction	2.19E-04	*ITGB4*, *DAG1*, *ITGA2*, *ITGB5*, *ITGA3*, *SDC4*, *ITGB1*, *HMMR*, *CD47*, *LAMB3*, *CD44*, *ITGA6*, *ITGAV*, *COL6A2*, *COL6A1*, *LAMC1*, *LAMB1*
Cell adhesion molecules (CAMs)	3.43E-04	*GLG1*, *F11R*, *CLDN7*, *MPZL1*, *PTPRF*, *HLA-A*, *CD99*, *CDH1*, *HLA-C*, *NEO1*, *HLA-B*, *CDH3*, *HLA-E*, *SDC4*, *ITGB1*, *HLA-G*, *HLA-F*, *ALCAM*, *ITGA6*, *ITGAV*, *PVRL3*, *CD58*, *VCAN*
Other glycan degradation	0.003983	*MAN2B2*, *HEXA*, *HEXB*, *MAN2B1*, *FUCA2*, *MANBA*
Sphingolipid metabolism	0.005227	*ACER3*, *SPTLC1*, *SGMS2*, *SPTLC2*, *PPAP2C*, *UGCG*, *KDSR*, *PPAP2A*, *PPAP2B*
Adherens junction	0.007454	*EGFR*, *PTPRF*, *ACTN4*, *TGFBR1*, *MET*, *ACTN1*, *CTNND1*, *CDH1*, *FER*, *CTNNA1*, *IQGAP1*, *VCL*, *PVRL3*
Heparan sulfate biosynthesis	0.008368	*NDST1*, *HS3ST1*, *HS6ST2*, *EXT1*, *EXT2*, *GLCE*, *HS2ST1*
Glycosaminoglycan degradation	0.01383	*GNS*, *IDS*, *HPSE*, *HEXA*, *GUSB*, *HEXB*
Biosynthesis of unsaturated fatty acids	0.016882	*PTPLB*, *ELOVL5*, *FADS1*, *SCD*, *HSD17B12*, *SCD5*
Steroid biosynthesis	0.028381	*SOAT1*, *LIPA*, *DHCR7*, *SC5DL*, *DHCR24*
Regulation of actin cytoskeleton	0.029937	*EGFR*, *ACTN4*, *DIAPH1*, *DIAPH2*, *DIAPH3*, *IQGAP3*, *ITGB4*, *ITGA2*, *ACTN1*, *ITGB5*, *RDX*, *IGF2*, *ITGA3*, *MYH9*, *ITGB1*, *IQGAP1*, *VCL*, *EZR*, *ITGA6*, *ITGAV*, *PPP1R12A*, *MSN*, *MYH10*, *F2R*
Spliceosome	0.032223	*HSPA1A*, *SNW1*, *CDC5L*, *SF3B3*, *HNRNPA3*, *HNRNPM*, *SF3B1*, *TCERG1*, *AQR*, *PRPF8*, *CDC40*, *SNRNP200*, *ACIN1*, *THOC2*, *RBM25*, *PRPF40A*
Ether lipid metabolism	0.034476	*PLA2G4A*, *PPAP2C*, *LCLAT1*, *PPAP2A*, *PPAP2B*, *CHPT1*, *AGPAT2*
Focal adhesion	0.046005	*EGFR*, *ACTN4*, *DIAPH1*, *MET*, *ITGB4*, *ITGA2*, *ACTN1*, *ITGB5*, *ITGA3*, *ITGB1*, *FLNB*, *VCL*, *LAMB3*, *ITGA6*, *JUN*, *ITGAV*, *VEGFA*, *COL6A2*, *PPP1R12A*, *COL6A1*, *LAMC1*, *LAMB1*
Glycerophospholipid metabolism	0.049458	*CDS2*, *PLA2G4A*, *PPAP2C*, *LCLAT1*, *PPAP2A*, *CDS1*, *PTDSS1*, *PPAP2B*, *CHPT1*, *AGPAT2*

**Table 2:** Functional classification of the translationally down-regulated genes in HCT116 cells exposed to hypoxia for 16 h.

KEGG_pathway	P-value	Genes
Apoptosis	0.006497055	*CASP6*, *PRKAR2B*, *CASP7*, *BAX*, *CHP*, *CHUK*, *PRKX*, *TRADD*
Ubiquitin mediated proteolysis	0.008019849	*UBE2N*, *UBE2D2*, *ANAPC5*, *SKP2*, *UBE2L6*, *PIAS2*, *SKP1*, *UBE3C*, *UBE2B*, *STUB1*
Oxidative phosphorylation	0.04791609	*ATP5D*, *ATP6V1C1*, *NDUFB10*, *NDUFB7*, *NDUFS8*, *ATP5G3*, *NDUFB1*, *NDUFB2*

### Hypoxia induces lysosomal autophagy and mitochondrial dysfunction through translational regulation in HCT116 cells

**Fig 4:**
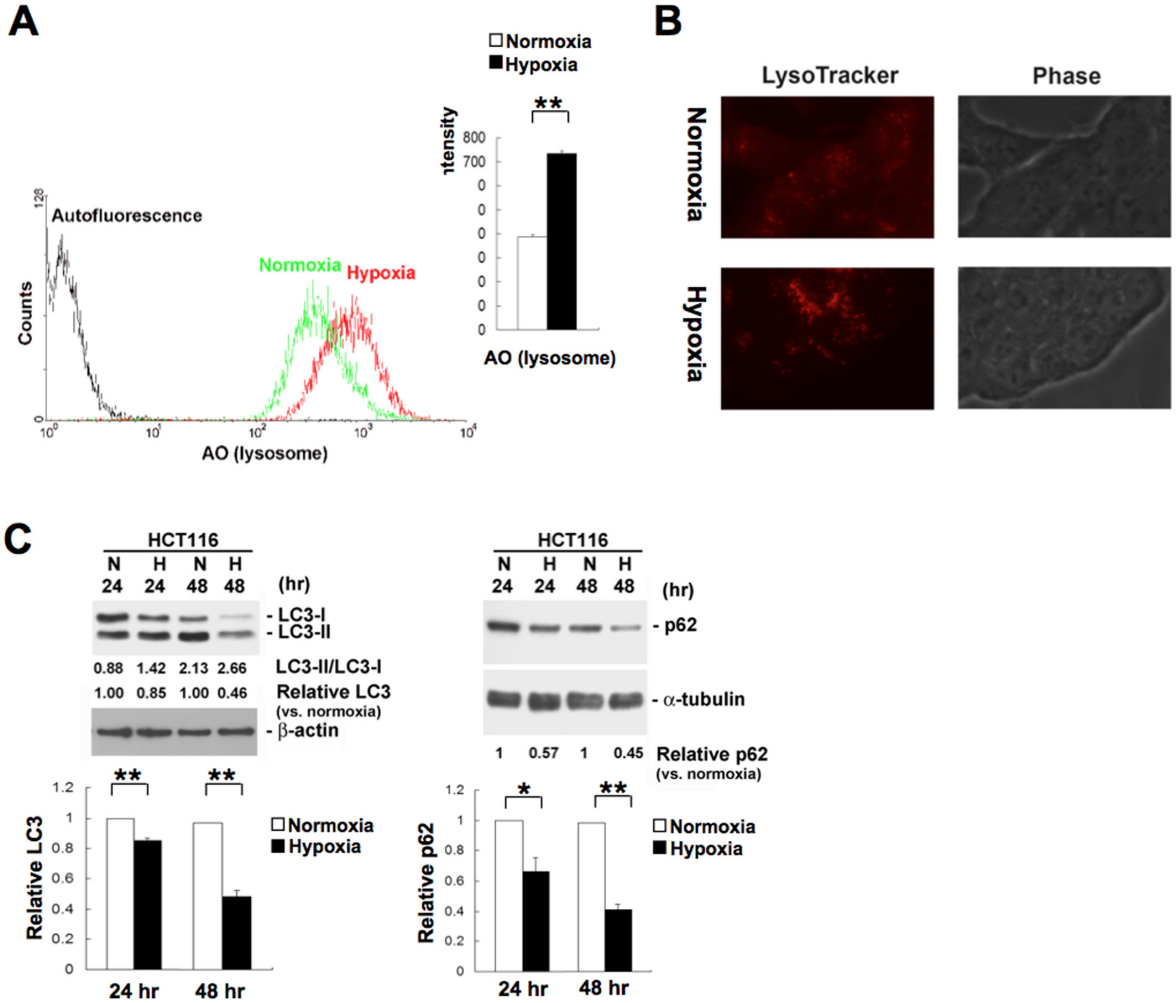
Hypoxia induces autophagy and the enrichment of lysosomes in HCT116 cells. **A**. HCT116 cells were grown under normoxia (21% O_2_) or hypoxia (1% O_2_) for 24 h. Cells were stained with acridine orange (AO) and analyzed by flow cytometry to measure the content of lysosomes. Bar graphs show mean fluorescence intensity of AO from at least three independent experiments (**p < 0.01). **B**. HCT116 cells were grown under normoxia (21% O_2_) or hypoxia (1% O_2_) for 24 h. Lysosomes were labeled with LysoTracker Red DND-99 for 1 h in living cells and observed by an inverted fluorescence microscope. **C**. HCT116 cells were exposed to hypoxia (H) or normoxia (N) for 24 h and 48 h. Total cell extracts were analyzed by immunoblotting with LC3 and β-actin antibodies (left panel). The LC3-I and LC3-II bands were quantified, and autophagy was measured by variations in the ratio of LC3-II/LC3-I and the total amount of LC3 (LC3-I plus LC3-II) normalized to β-actin for each condition. Bar graphs show relative LC3 protein level normalized to β-actin from at least three independent experiments (**p < 0.01). The above samples were also analyzed by immunoblotting with p62 and α-tubulin antibodies (right panel). The protein level of p62 relative to α-tubulin was quantified. Bar graphs show relative p62 protein level normalized to α-tubulin from at least three independent experiments (*p < 0.05, **p < 0.01).

**Fig 5:**
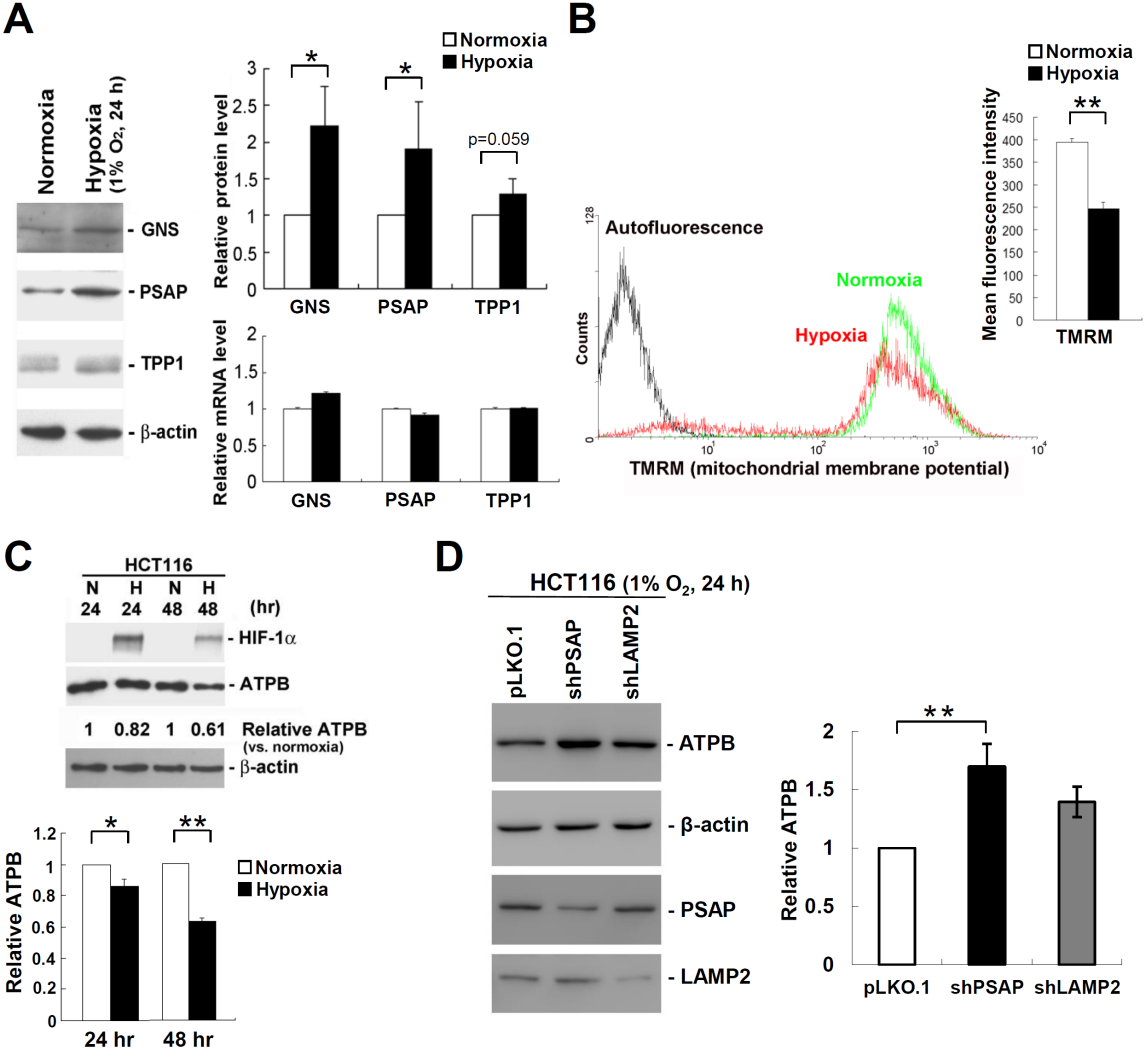
Hypoxia induces mitochondrial autophagy through translational regulation of lysosomal proteins. **A**. HCT116 cells were exposed to hypoxia or normoxia for 24 h. The levels of lysosomal proteins glucosamine (N-acetyl)-6-sulfatase (GNS), prosaposin (PSAP), and tripeptidyl peptidase 1 (TPP1) were detected by immunoblot analysis. The levels of mRNA expression were analyzed by quantitative real-time RT-PCR. Detection of β-actin protein and mRNA served as loading controls. Bar graphs show relative protein (upper) and mRNA (lower) levels normalized to β-actin from at least three independent experiments (*p < 0.05). **B**. HCT116 cells were grown under hypoxia or normoxia for 24 h. Cells were stained with tetramethylrhodamine methyl ester (TMRM) and analyzed by flow cytometry to detect the membrane potential of mitochondria. Bar graphs show mean fluorescence intensity of TMRM from at least three independent experiments (**p < 0.01). **C**. HCT116 cells were exposed to hypoxia (H) or normoxia (N) for 24 h and 48 h. Total cell extracts were analyzed by immunoblotting with HIF-1α, ATPB, and β-actin antibodies. The protein level of ATPB relative to β-actin was quantified. Bar graphs show relative ATPB protein level normalized to β-actin from at least three independent experiments (*p < 0.05, **p < 0.01). **D**. HCT116 cells were transfected with the empty vector (pLKO.1) or the pLKO.1 vector expressing the indicated shRNAs (shPSAP and shLAMP2). At 2 days post-transfection, cells were treated with hypoxia (1% O_2_) for 24 h. Immunoblotting was performed using antibodies against ATPB, β-actin, PSAP, and LAMP2. The protein level of ATPB relative to β-actin was quantified. Bar graphs show relative ATPB protein level normalized to β-actin from at least three independent experiments (**p < 0.01).

We have identified 35 translationally up-regulated genes that function in the lysosomal pathway in HCT116 cells exposed to hypoxia (Table 1). Interestingly, all 35 lysosomal genes were up-regulated during hypoxia at the translational level independently of transcription (Table 3). This indicates that translational regulation may play a crucial role in hypoxia-induced autophagy. Lysosomes can be quantified by flow cytometry after staining the cells with acridine orange (AO), a lysosomotropic weak base that accumulates within the acidic vesicular organelles of living cells. The fluorescence intensity of AO was significantly increased in HCT116 cells after exposure to hypoxia for 24 h (Fig 4A), suggesting that hypoxia leads to an increase in the content of lysosomes. We next used LysoTracker Red DND-99, a fluorescent acidotropic dye for labeling and tracking acidic organelles in living cells, to stain lysosomes. Lysosomes were stained bright red in HCT116 cells, and hypoxia gives rise to enlarged lysosomes as compared to normoxia (Fig 4B). The results indicate that hypoxia increases the size of lysosomal volume. We further examined the induction of autophagy by monitoring the autophagy marker protein light chain 3 (LC3) in HCT116 cells under hypoxia compared to normoxia. Conversion of LC3-I to LC3-II and degradation of total LC3 (LC3-I plus LC3-II) were used to determine changes in the extent of autophagy [41]. Immunoblot analysis showed that LC3-I to LC3-II conversion (LC3-II/LC3-I ratio) was increased and the total amount of LC3 was decreased in HCT116 cells exposed to hypoxia for 24 h and 48 h (Fig 4C, left panel), suggesting hypoxia-induced autophagy. We also detected the autophagy marker protein p62, which is degraded during autophagy [41]. Consistently, the amount of p62 also showed a marked decrease in hypoxic HCT116 cells (Fig 4C, right panel). To verify hypoxia-induced translational up-regulation of lysosomal proteins (Table 3), we detected both the protein and mRNA levels of lysosomal genes glucosamine (N-acetyl)-6-sulfatase (*GNS*), prosaposin (*PSAP*), and tripeptidyl peptidase 1 (*TPP1*). After exposure to hypoxia for 24 h, the level of GNS and PSAP proteins was increased by ~2-fold as compared to normoxia (Fig 5A). The level of TPP1 protein was also slightly increased during hypoxia. A quantitative assay showed that the mRNA levels of GNS, PSAP, and TPP1 are not significantly affected by hypoxia (Fig 5A). The results indicate that hypoxia enriches lysosomal proteins through translational mechanisms.

**Table 3:** Translationally up-regulated genes involved in lysosome in HCT116 cells exposed to hypoxia for 16 h.

Gene symbol	Gene name	Polysomal RNA	Total RNA	Polysomal/total	GenBank
*GNS*	glucosamine (N-acetyl)-6-sulfatase	15.17	1.14	13.31	NM_002076
*PSAP*	prosaposin	10.04	0.95	10.57	NM_002778
*TPP1*	tripeptidyl peptidase I	9.95	1.03	9.66	NM_000391
*HEXB*	hexosaminidase B (beta polypeptide)	11.05	1.2	9.21	NM_000521
*ATP6AP1*	ATPase, H+ transporting, lysosomal accessory protein 1	9.47	1.18	8.03	NM_001183
*LAMP2*	lysosomal-associated membrane protein 2	10.37	1.3	7.98	NM_013995
*LGMN*	legumain	8.23	1.07	7.69	NM_001164692
*CTSA*	cathepsin A	7.41	1.01	7.34	NM_017573
*CTSB*	cathepsin B	7.15	1	7.15	NM_001908
*LIPA*	lipase A, lysosomal acid, cholesterol esterase	5.67	0.8	7.09	NM_000235
*LAMP1*	lysosomal-associated membrane protein 1	7.88	1.16	6.79	NM_005561
*PPT1*	palmitoyl-protein thioesterase 1	7.47	1.12	6.67	NM_000310
*NPC1*	Niemann-Pick disease, type C1	5.83	0.93	6.27	NM_000271
*CD164*	CD164 molecule, sialomucin	6.02	0.99	6.08	NM_003820
*LAPTM4A*	lysosomal protein transmembrane 4 alpha	5.38	0.91	5.91	NM_014713
*CD63*	CD63 molecule	4.78	0.86	5.56	NM_014652
*LAPTM4B*	lysosomal protein transmembrane 4 beta	4.71	0.91	5.18	NM_018407
*GUSB*	glucuronidase, beta	6.97	1.4	4.98	NM_000181
*CTSL2*	cathepsin L2	4.97	1.02	4.87	NM_001201575
*SORT1*	sortilin 1	3.55	0.85	4.18	NM_005978
*SCARB2*	scavenger receptor class B, member 2	3.49	0.91	3.84	NM_001204255
*CLN5*	ceroid-lipofuscinosis, neuronal 5	5.21	1.43	3.64	NM_006493
*AP1G1*	adaptor-related protein complex 1, gamma 1 subunit	3.72	1.16	3.21	NM_001030007
*MAN2B1*	mannosidase, alpha, class 2B, member 1	4.65	1.48	3.14	NM_000528
*CTSC*	cathepsin C	3.4	1.09	3.12	NM_148170
*MANBA*	mannosidase, beta A, lysosomal	4.34	1.4	3.1	NM_005908
*CLTC*	clathrin, heavy chain (Hc)	2.75	0.92	2.99	NM_004859
*CTSL1*	cathepsin L1	2.77	0.97	2.86	NM_145918
*M6PR*	mannose-6-phosphate receptor (cation dependent)	2.56	0.9	2.84	NM_005898
*IDS*	iduronate 2-sulfatase	3.79	1.4	2.71	NM_000202
*GAA*	glucosidase, alpha; acid	3.47	1.29	2.69	NM_000152
*HEXA*	hexosaminidase A (alpha polypeptide)	3.21	1.29	2.49	NM_000520
*ATP6V0B*	ATPase, H+ transporting, lysosomal 21kDa, V0 subunit b	2.41	1.06	2.27	NM_004573
*CTSH*	cathepsin H	2.31	1.04	2.22	NM_004390
*AP3B1*	adaptor-related protein complex 3, beta 1 subunit	2.91	1.35	2.16	NM_053042

Clearance of damaged mitochondria by autophagy, a process also called mitophagy, prevents the accumulation of reactive oxygen species (ROS) during hypoxia. We also identified 22 translationally down-regulated genes involved in mitochondrial functions in HCT116 cells (Table 4). Evidently, hypoxia represses translation of genes involved in oxidative phosphorylation (*ATP5D*, *ATP6V1C1*, *NDUFB10*, *NDUFB7*, *NDUFS8*, *ATP5G3*, *NDUFB1*, and *NDUFB2*), mitochondrial ribosomal proteins (*MRPL36*, *MRPL12*, *MRP63*, *MRPL41*, *MRPS7*, *MRPS34*, *MRPS16*, *MRPL43*, *MRPL34*, *MRPS12*, *MRPL38*, and *MRPS26*), and mitochondrial transport (*TIMM10* and *TOMM40L*). The cationic dye tetramethylrhodamine methyl ester (TMRM) was used to assess mitochondrial membrane potential changes in living cells. The fluorescence intensity of TMRM was significantly decreased in HCT116 cells exposed to hypoxia for 24 h (Fig 5B), suggesting hypoxia-induced mitochondrial dysfunction. In addition to flow cytometry study of mitochondrial function, we also detected the protein level of mitochondrial membrane ATP synthase beta subunit (ATPB), which produces ATP from ADP in the presence of a proton gradient across the inner membrane of mitochondria. Immunoblot analysis showed that the amount of ATPB protein was decreased in HCT116 cells exposed to hypoxia for 24 h and 48 h (Fig 5C). This may be due to mitophagy and translational repression of mitochondrial proteins. We further performed RNAi-mediated knockdown to silence *PSAP* and *LAMP2* genes whose translation is up-regulated during hypoxia in HCT116 cells (Table 3) and then evaluate its influence on mitophagy. Interestingly, we observed that knockdown of *PSAP* and *LAMP2* genes increases ATPB abundance during hypoxia in HCT116 cells (Fig 5D). The results indicate that PSAP and LAMP2 proteins may play a key role in mitophagy during hypoxia. Consistent with the proposition, translational regulation of lysosomal proteins may play an important role in autophagy during hypoxia.

**Table 4:** Translationally down-regulated genes involved in mitochondrial functions in HCT116 cells exposed to hypoxia for 16 h.

Gene symbol	Gene name	Polysomal RNA	Total RNA	Polysomal /total	GenBank
*MTFMT*	Mitochondrial methionyl-tRNA formyltransferase	0.22	0.73	0.3	NM_139242
*ATP5D*	ATP synthase, H+ transporting, mitochondrial F1 complex, delta subunit	0.31	0.96	0.32	NM_005017
*MPV17L2*	MPV17 mitochondrial membrane protein-like 2	0.25	0.75	0.33	NM_032683
*MRPL36*	mitochondrial ribosomal protein L36	0.28	0.82	0.34	NM_032479
*MRPL12*	mitochondrial ribosomal protein L12	0.3	0.87	0.34	NM_002949
*MRP63*	mitochondrial ribosomal protein 63	0.36	1.03	0.35	NM_024026
*MRPL41*	mitochondrial ribosomal protein L41	0.32	0.87	0.37	NM_032477
*TOMM40L*	translocase of outer mitochondrial membrane 40 homolog (yeast)-like	0.22	0.6	0.37	NM_032174
*MRPS7*	mitochondrial ribosomal protein S7	0.3	0.76	0.39	NM_015971
*MFF*	mitochondrial fission factor	0.4	1	0.4	NM_020195
*ATP5G3*	ATP synthase, H+ transporting, mitochondrial F0 complex, subunit C3 (subunit 9)	0.31	0.76	0.41	NM_001689
*MRPS34*	mitochondrial ribosomal protein S34	0.32	0.78	0.41	NM_023936
*MRPS16*	mitochondrial ribosomal protein S16	0.3	0.68	0.44	NM_016065
*SLC25A1*	solute carrier family 25 (mitochondrial carrier; citrate transporter), member 1	0.25	0.55	0.45	NM_005630
*MRPL43*	mitochondrial ribosomal protein L43	0.4	0.87	0.46	NM_032112
*MRPL34*	mitochondrial ribosomal protein L34	0.41	0.89	0.46	NM_023937
*SLC25A19*	solute carrier family 25 (mitochondrial thiamine pyrophosphate carrier), member 19	0.39	0.83	0.47	NM_021734
*MRPS12*	mitochondrial ribosomal protein S12	0.39	0.83	0.47	NM_021107
*COG8*	component of oligomeric golgi complex 8 (mitochondrial)	0.28	0.58	0.48	NM_022341
*MRPL38*	mitochondrial ribosomal protein L38	0.34	0.71	0.48	NM_032478
*TIMM10*	translocase of inner mitochondrial membrane 10 homolog (yeast)	0.43	0.87	0.49	NM_012456
*MRPS26*	mitochondrial ribosomal protein S26	0.4	0.8	0.5	NM_030811

### The abundance of several translation factors is regulated by hypoxia at the translational level

**Fig 6:**
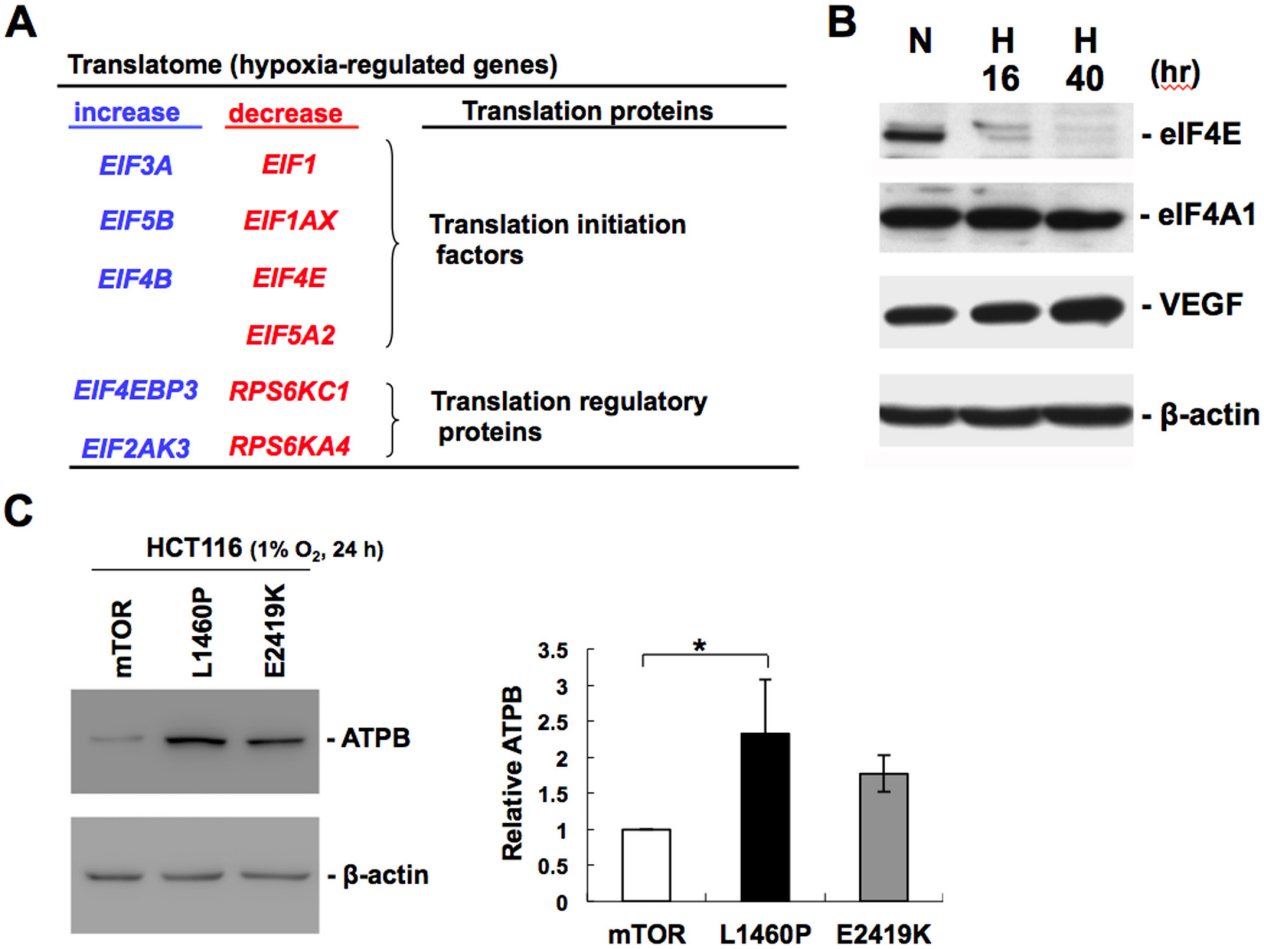
The abundance of several translation factors and the mTOR kinase activity are involved in hypoxia-induced autophagy in HCT116 cells. **A**. List of hypoxia-regulated genes that function as translation initiation factors and translation regulatory proteins in HCT116 cells. The color represents up-regulated (blue) or down-regulated (red) genes at the translational level. **B**. HCT116 cells were exposed to 16 h and 40 h of hypoxia (H) compared to that of cells in normoxia (N). Immunoblot analysis of eIF4E, eIF4A1, VEGFA, and β-actin proteins in HCT116 cells exposed to hypoxia for the indicated period of time. Detection of β-actin protein served as a loading control. **C**. HCT116 cells were transfected with the vector expressing wild type mTOR or constitutively active mutants (L1460P & E2419K). At 24 h post-transfection, cells were treated with hypoxia (1% O_2_) for 24 h. Immunoblotting was performed using antibodies against ATPB and β-actin. The protein level of ATPB relative to β-actin was quantified. Bar graphs show relative ATPB protein level normalized to β-actin from at least three independent experiments (*p < 0.05).

Among these hypoxia-regulated candidate genes (Fig 2), we found that several translation initiation factors and translation regulatory proteins were susceptible to hypoxia at the translational level (Fig 6A). Notably, these hypoxia-regulated translation factors are also involved in PERK and mTOR signaling pathways. Microarray data showed that hypoxia up-regulated the translation of *EIF4EBP3* and *EIF2AK3* (also known as *PERK*), whereas translation of *EIF4E* and RPS6K subunits (*RPS6KC1* and *RPS6KA4*) was down-regulated by hypoxia in HCT116 cells (Fig 6A). We also performed immunoblotting to verify translational down-regulation of eIF4E in hypoxic HCT116 cells. Immunoblot analysis showed that the abundance of eIF4E protein was evidently decreased in HCT116 cells exposed to hypoxia for 16 h and 40 h (Fig 6B), whereas the abundance of translation initiation factor eIF4A1 was not significantly affected by hypoxia. In contrast, the expression of hypoxia-responsive VEGF protein was gradually increased in HCT116 cells under hypoxia (Fig 6B).

### The mTOR kinase activity is involved in hypoxia-induced autophagy in HCT116 cells

**Fig 7:**
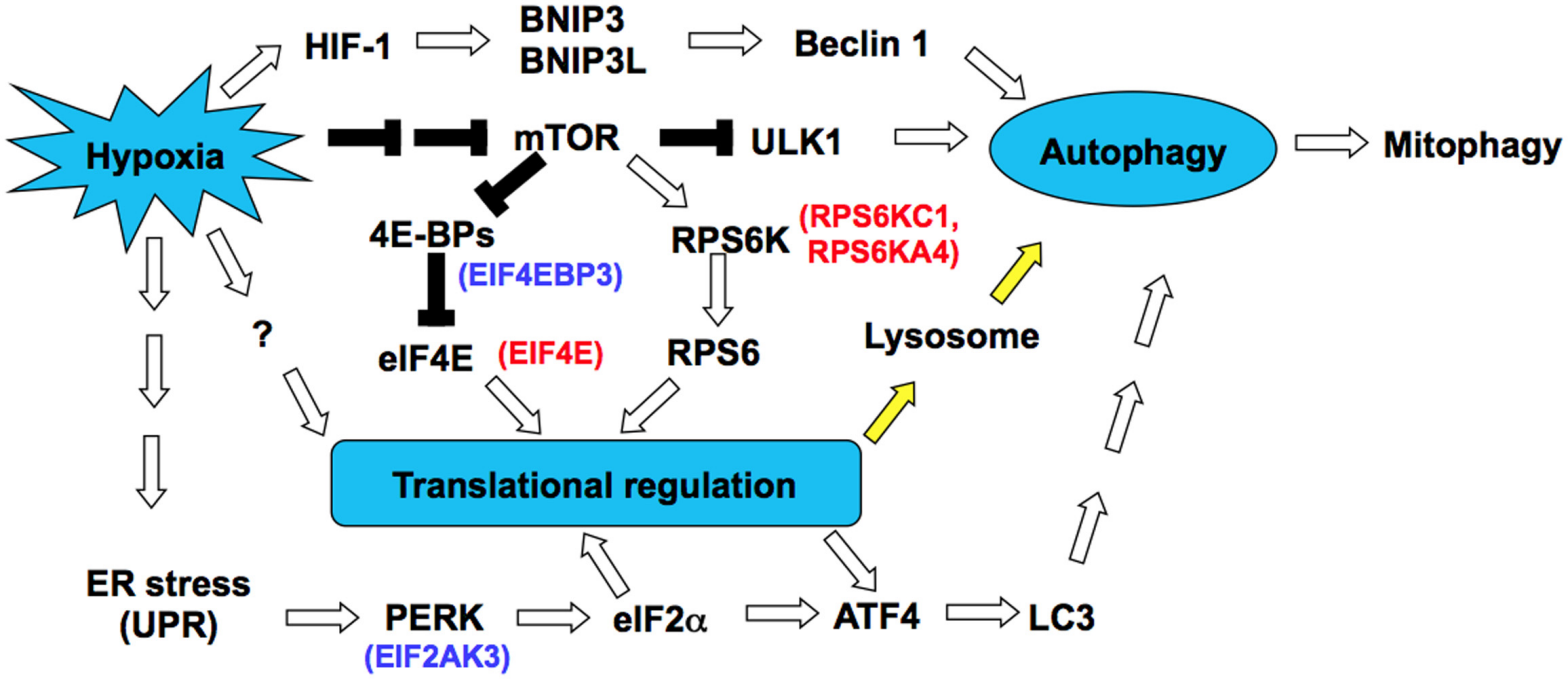
A simple model of hypoxia-induced autophagy in HCT116 cells. Hypoxia-inducible factor HIF-1 promotes *BNIP3* and *BNIP3L* transcription, thereby activating Beclin 1 by disrupting the Bcl-2-Beclin1 complex. Beclin 1 is required for the nucleation of autophagy. The mTOR signaling pathway plays a central role in hypoxia-induced autophagy. Inactivation of mTOR during hypoxia leads to activation of the autophagy-initiating kinase ULK1, which is required for the initiation of autophagy. Translational regulation also plays provital roles in hypoxia-induced autophagy, including mitochondrial autophagy (Mitophagy). Hypoxia inactivates mTOR and thus leads to dephosphorylation of 4E-BPs, which represses cap-dependent translation initiation by sequestering eIF4E. The RPS6 kinase RPS6K is also down-regulated by mTOR inactivation. On the other hand, hypoxia causes ER stress and thus leads to phosphorylation of eIF2α by PERK. Phosphorylation of eIF2α inhibits translation initiation by preventing the eIF2-GTP-tRNA(i)Met ternary complex formation. Other unknown mechanisms (?) of translational regulation during hypoxia remain to be established. Notably, hypoxia induces translation of lysosomal proteins (yellow arrow) and ATF4, which activates LC3-dependent autophagy. The abundance of several translation factors involved in the mTOR and PERK signaling pathways is also regulated by hypoxia. The color represents up-regulated (blue) or down-regulated (red) genes at the translational level.

Furthermore, we overexpressed constitutively active mutants of mTOR (L1460P & E2419K) in HCT116 cells to determine whether the mTOR kinase activity can prevent hypoxia-induced autophagy. As expected, constitutively active mTOR mutants increase ATPB abundance during hypoxia in HCT116 cells as compared to wild type mTOR (Fig 6C). This indicates that constitutively active mutants of mTOR can prevent hypoxia-induced mitophagy, supporting the role of mTOR kinase activity in regulating autophagy during hypoxia. Here, we propose a model of hypoxia-induced autophagy in HCT116 cells (Fig 7). It has been reported that hypoxia induces autophagy in a HIF-1-dependent manner [36, 37]. HIF-1 promotes the transcription of BNIP3 and BNIP3L [36], two BH3-only proteins that activates Beclin 1 for the nucleation of autophagosome. Consistent with previous studies [36, 37], we also observed that BNIP3 and BNIP3L were transcriptionally up-regulated by 6.45 and 4.04 folds in HCT116 cells during hypoxia. In addition, autophagy is regulated by the mTOR signaling pathway, which negatively regulates unc-51 like autophagy activating kinase 1 (ULK1) [42]. Inactivation of mTOR induces autophagy through activation of ULK1 [43, 44]. Importantly, mTOR also regulates translation through its downstream substrates 4E-BPs and RPS6K, which regulate the activities of translation factors eIF4E and RPS6, respectively. On the other hand, the eIF2α kinase PERK also plays a key role in the formation of autophagy [45]. Hypoxia induces ER stress that leads to the UPR and activates PERK. PERK regulates translation by phosphorylating eIF2α, and this up-regulates ATF4 to activate LC3-dependent autophagy during hypoxia. Moreover, the abundance of several translation factors involved in the mTOR and PERK signaling pathways is also controlled by hypoxia (Fig 7). We herein provide experimental evidence that hypoxia promotes lysosomal autophagy through translational regulation (Fig 7, yellow arrow); however the molecular details of hypoxia-induced translation remain mostly unresolved. In summary, hypoxia may induce autophagy through HIF-1-mediated transcription in cooperation with signal transduction and translational regulation in HCT116 cells.

## Discussion

Translational regulation provides a rapid and reversible mechanism for cell adaptation to hypoxia, and it varies a lot depending on the hypoxic conditions and cell types. We utilized whole human genome microarray to identify hypoxia-regulated genes in HCT116 cells. Microarray data showed that hypoxia lead to the translational changes of 1,516 genes and the transcriptional changes of 278 genes in HCT116 cells (Fig 2). Many HIF-1 target genes, such as *ALDOA*, *ALDOC*, *ENO1*, *GLUT1*, *GLUT3*, *HK2*, *PDK1*, *VEGFA*, and *ADM*, were identified as transcriptionally up-regulated genes in HCT116 cells during hypoxia (data not shown). This indicates that HIF-1-mediated transcriptional regulation plays a key role in cellular adaptation to hypoxia. Enhanced glycolysis and angiogenesis are the most common characteristics of tumor cells undergoing hypoxia [46]. We also observed that both transcriptional and translational up-regulation of genes include glucose transporters (*GLUT1* and *GLUT3*), glycolytic enzyme (*HK2*), and angiogenic factors (*VEGFA* and *CYR61*) in hypoxic HCT116 cells. Cell proliferation is frequently impaired in mammalian cultured cells during hypoxia [47, 48]. Although the molecular mechanisms of down-regulated genes at both the transcriptional and translational levels remain largely unknown, we also found that both transcriptional and translational down-regulation of S-phase kinase-associated protein 2 (*SKP2*) and cyclin E1 (*CCNE1*), which may account for hypoxia-induced G1 phase arrest of the cell cycle [48]. Pathway enrichment analysis shows that hypoxia-regulated genes are involved in a variety of biological processes (Tables 1 & 2). Hypoxia up-regulates translation of genes involved in lysosome and metabolic pathways, antigen processing and presentation, cell adhesion and migration (Table 1), whereas genes involved in apoptosis, protein degradation, and oxidative phosphorylation are down-regulated by hypoxia in HCT116 cells (Table 2). Autophagy is an adaptive metabolic response to maintain energy homeostasis under nutrient deprivation [49]. Hypoxia-induced autophagy is an important cytoprotective and drug resistance mechanism that may promote tumor cell survival. Recent studies have shown that antitumor efficacy of antiangiogenic therapy is synergistically enhanced via combined autophagy inhibition in glioma [50, 51]. It is therefore important to understand the regulation of autophagy for improving anticancer therapy. Here, we provide evidence that hypoxia activates autophagy through translational up-regulation of many lysosomal proteins (Fig 5A & Table 3). We also demonstrate that knockdown of lysosomal proteins (PSAP and LAMP2) increases ATPB abundance during hypoxia in HCT116 cells (Fig 5D). Both PSAP and LAMP2 are highly conserved glycoproteins whose translation is up-regulated during hypoxia in HCT116 cells (Table 3). PSAP is required for the hydrolysis of glycosphingolipids in lysosomes, whereas LAMP2 is shown to participate in lysosomal biogenesis. Notably, autophagy is impaired in mice with deficiency of *PSAP* [52] or *LAMP2* [53], due to defects in lysosomal degradation. Their findings are consistent with the proposition that translational regulation of lysosomal proteins plays an important role in hypoxia-induced autophagy.

It has been reported that hypoxia-induced autophagy enhances antigen presentation of inflammatory response [54]. Consistent with this notion, we also identified many translationally up-regulated genes that function in antigen processing and presentation (Table 1), suggesting a state of chronic inflammation in cells exposed to hypoxia [54, 55]. It is thought that tumor cells adapt to hypoxia through a complex metabolic rearrangement [56]. In addition to glycolysis, we observed that many translationally up-regulated genes are involved in the metabolism of glycan and lipids in hypoxic HCT116 cells (Table 1). Hypoxia is associated with poor prognosis in cancer patients since hypoxic regions within tumors are resistant to apoptosis [57]. We also observed that hypoxia down-regulates translation of apoptosis-related genes, such as *CASP6*, *PRKAR2B*, *CASP7*, *BAX*, *CHP*, *CHUK*, *PRKX*, and *TRADD* (Table 2), and this may result in apoptotic resistance in hypoxic HCT116 cells. HIF-1α protein is constantly synthesized, but rapidly degraded by oxygen-dependent hydroxylation and ubiquitin-mediated proteolysis under normoxia [58]. Several genes involved in ubiquitin-mediated proteolysis were down-regulated by hypoxia at the translational level (Table 2), suggesting that translational regulation also contributes to the increased stability of HIF-1α protein. Mitochondria are the major consumers of cellular oxygen within cells. Many mitochondrial genes were down-regulated by hypoxia at the translational level (Table 4). Notably, 12 mitochondria-specific ribosomal proteins (*MRPL36*, *MRPL12*, *MRP63*, *MRPL41*, *MRPS7*, *MRPS34*, *MRPS16*, *MRPL43*, *MRPL34*, *MRPS12*, *MRPL38*, and *MRPS26*) are more susceptible to translational repression during hypoxia. It would be interesting to explore the molecular basis of translational down-regulation of mitochondrial ribosomal proteins in hypoxic HCT116 cells. Hypoxia has been shown to cause a decrease in mitochondrial biogenesis and cellular respiration by down-regulation of c-Myc protein expression in renal carcinoma cells [59]. Therefore, mitochondrial dysfunction should be a common event in tumor cells exposed to hypoxia.

Protein synthesis is a major consumer of cellular ATP. Inhibition of general translation is required to maintain energy homeostasis during hypoxia. We have shown that hypoxia inhibits general translation through activation of the PERK signaling pathway and inactivation of the mTOR signaling pathway in HCT116 cells (Fig 1C). However, the molecular details of hypoxia-induced translational activation have not been well elucidated. More recently, we demonstrated that the expression of human fibroblast growth factor 9 (FGF9) is up-regulated by hypoxia through IRES-mediated translation [60]. According to the UTRdb (http://utrdb.ba.itb.cnr.it/), a database of 5’ and 3’ UTRs of eukaryotic mRNAs, a total of 27,325 human mRNAs are predicted to contain 6,535 IRES (~23.9% in average) in their 5’ UTRs. The analysis of translatome data showed that ~32.4% of up-regulated genes express IRES-containing mRNAs in hypoxic HCT116 cells. This indicates that IRES-mediated translation may contribute, at least in part, to hypoxia-induced gene expression in human colon cancer cells. However, alternative mechanisms by which hypoxia activates translation of selected mRNAs remain to be further investigated.

## Supporting Information

S1 Fig Hypoxia dramatically inhibits translation of β-actin mRNA in HCT116 cells. Colorectal cancer cell lines HCT116, HT-29, Caco-2, and LS123 were treated with hypoxia (1% O_2_) for 24 h. The polysomal distribution of β-actin mRNA was detected by polysome profiling and RT-PCR. Translational efficiency of β-actin mRNA was calculated and shown as a percentage.
(PDF)

S1 Table List of primers used for qRT-PCR (FP: forward primer; RP: reverse primer). (PDF)
