# Supplementary figures and images for: Hypoxia Induces Autophagy through Translational Up-Regulation of Lysosomal Proteins in Human Colon Cancer Cells
Source: PLoS One. 2016 Apr 14;11(4):e0153627. doi: 10.1371/journal.pone.0153627 (PMC4831676; doi:10.1371/journal.pone.0153627)

# S1 Fig

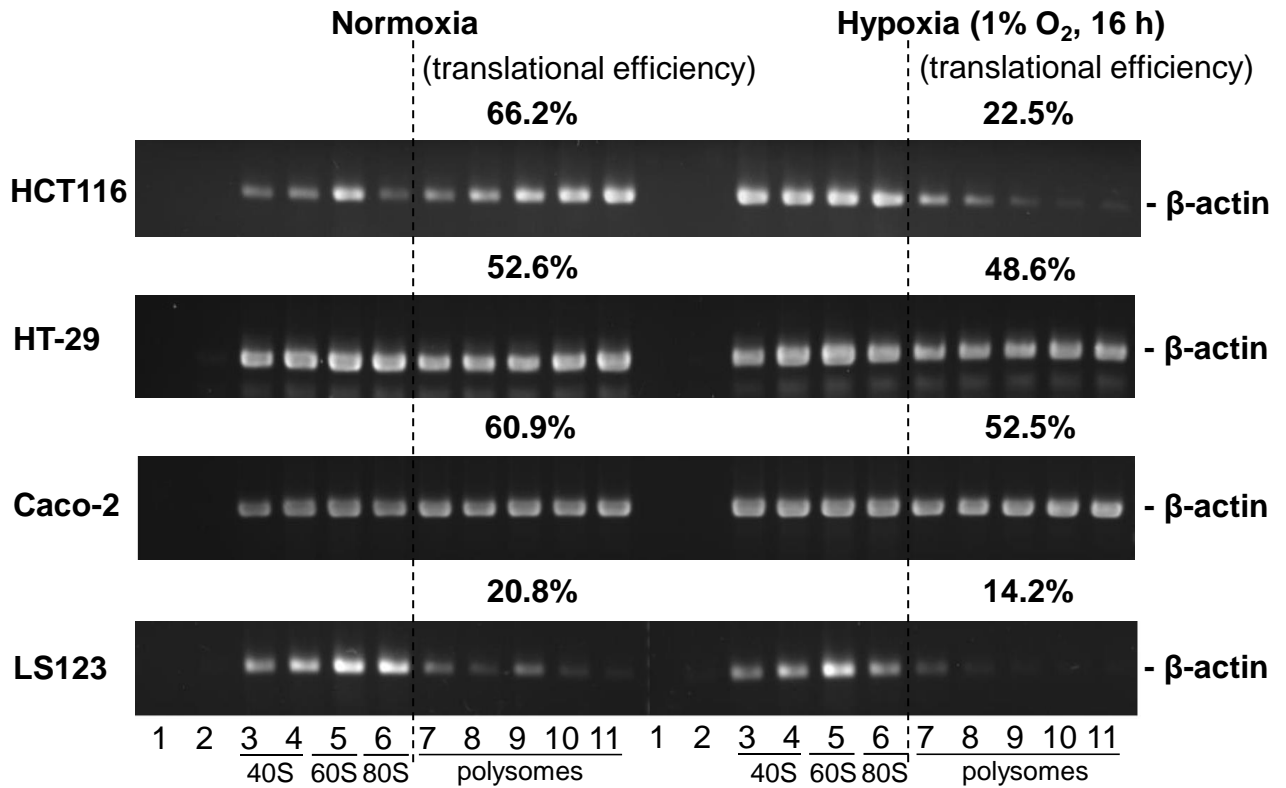

Supplement: S1 Fig — Colorectal cancer cell lines HCT116, HT-29, Caco-2, and LS123 were treated with hypoxia (1% O2) for 24 h. The polysomal distribution of β-actin mRNA was detected by polysome profiling and RT-PCR. Translational efficiency of β-actin mRNA was calculated and shown as a percentage. (PDF) [file pone.0153627.s001.pdf]
