# Supplementary material for: Hypoxia Induces Autophagy through Translational Up-Regulation of Lysosomal Proteins in Human Colon Cancer Cells
Source: PLoS One. 2016 Apr 14;11(4):e0153627. doi: 10.1371/journal.pone.0153627 (PMC4831676; doi:10.1371/journal.pone.0153627)
Supplement: S1 Table — (PDF) [file pone.0153627.s002.pdf]

S1 Table

Gene symbol	Primer
ACTB	FP: 5’GCCCTGAGGCACTCTTCCA3’
	RP: 5’CGGATGTCCACGTCACACTT3’
GLUT1	FP: 5’CTTCACTGTCGTGTCGCTGT3’
	RP: 5’TGAAGAGTTCAGCCACGATG3’
ADM	FP: 5’CGTCGGAGTTTCGAAAGAAG3’
	RP: 5’CCCTGGAAGTTGTTCATGCT3’
VEGFA	FP:5’CCTGGTGGACATCTTCCAGGAGTACC3’
	RP:5’GAAGCTCATCTCTCCTATGTGCTGGC3’
HSPA5	FP: 5’TAGCGTATGGTGCTGCTGTC3’
	RP: 5’TTTGTCAGGGGTCTTTCACC3’
VCAN	FP: 5’GGTGCACTTTGTGAGCAAGA3’
	RP: 5’TTCGTGAGACAGGATGCTTG3’
GPR126	FP: 5’GCGAGCAGAAACAACAATGA3’
	RP: 5’TTGCTTCTCTTGCCATTCCT3’
GNS	FP: 5’ GTTCGAGGACCTGGGATCAA3’
	RP: 5’ TTAGGTCGTAGCCAGCAATGTC3’
PSAP	FP: 5’ CTGCAGACCGTTTGGAACAAG3’
	RP: 5’ TGGCATTGTCCTTCAGCATATC3’
TPP1	FP: 5’ TGCCACCATCCAGTTACTTCAA3’
	RP: 5’ GGAATGGGCACTCTGTTGCT3’
